# Complete genome sequence of *Serratia plymuthica* SWSY-3.47

**DOI:** 10.1128/mra.00815-24

**Published:** 2024-10-14

**Authors:** Himari Takahashi, Tomoro Warashina, Yui Takahashi, Mayuki Tanaka, Kazushi Suzuki, Teppei Morita, Kazuharu Arakawa

**Affiliations:** 1 Institute for Advanced Biosciences, Keio University, Tsuruoka, Yamagata, Japan; 2 Faculty of Environment and Information Studies, Keio University, Fujisawa, Kanagawa, Japan; 3 Systems Biology Program, Graduate School of Media and Governance, Keio University, Fujisawa, Kanagawa, Japan; 4 Graduate School of Science and Technology, Niigata University, Niigata, Japan; 5 Faculty of Agriculture, Niigata University, Niigata, Japan; University of Strathclyde, Glasgow, United Kingdom

**Keywords:** *Serratia plymuthica* SWSY-3.47, complete genome, nanopore sequencing, chitinolytic activity

## Abstract

*Serratia plymuthica* strain SWSY-3.47 is a Gram-negative, rod-shaped bacteria isolated for its high chitinolytic activity. Here, we report the complete genome of this strain comprised of a single circular chromosome of 5,636,345 bp with a G + C content of 56.0%.

## ANNOUNCEMENT

*Serratia plymuthica* strain SWSY-3.47 is a Gram-negative, facultatively anaerobic, rod-shaped, non-spore-forming bacteria that was first isolated in the water of a sand dune lake, Sakata, in Niigata, Japan, and was shown to possess higher chitinase activity than the reference chitinolytic bacterium *Serratia marcescens* (1). Isolate SWSY-3.47 was previously identified to be *Serratia plymuthica* by 16S rRNA gene sequencing (1). Since bacterial chitinases are effective in the digestion of chitinous fungal cell walls, these chitinolytic bacteria are believed to be applicable as biocontrol agents against agricultural phytopathogens.

Cells of *S. plymuthica* SWSY-3.47 stored as glycerol stock were grown at 30°C in an LB (Luria-Bertani) medium under aerobic conditions. Approximately 2.0  ×  10^9^ cells were collected, and the genomic DNA was purified using NucleoBond HMW DNA (Macherey-Nagel, Düren, Germany), following the manufacturer’s protocol. Long-read sequencing libraries were prepared and multiplexed using the Rapid Barcoding Kit (SQK-RBK114; Oxford Nanopore Technologies). Libraries were sequenced using a FLO-MIN114 Flowcell with GridION X5 device (GridION software release 23.07.12; Oxford Nanopore Technologies). Long reads were basecalled, demultiplexed, and trimmed for the adaptor sequences using guppy 6.5.7 (super accurate mode). The quality of the long reads was assessed using Nanoplot (2), resulting in a total of 606 Mbp consisting of 49,517 reads of N50 length 27,384 bp. Reads longer than 10  kb (500 Mbp, around ×100 coverage) were adapter-trimmed using Porechop v0.2.3 (3) and were then assembled using Canu version 2.2 (4). The assembly generated a single assembled chromosome, which was manually circularized by deleting the overlapping ends, following the overlapped coordinates specified by Canu. The completeness statistics reached a maximum value by a single round of error correction using medaka v.1.11.3 with the reads after adapter trimming and did not show any improvement in subsequent rounds of error correction. The completeness of the assembly was assessed with CheckM version 1.2.2 (5) to be 99.98% with automatic lineage identification [f__Enterobacteriaceae (UID5066)]. The circular genome was annotated using the DDBJ Fast Annotation and Submission Tool (DFAST) version 1.2.18 (6) and was rotated so that *dnaA* gene was located at the start position. All software was used with their default settings unless otherwise specified.

The assembled genome had a total length of 5,636,345  bp and a G + C content of 56.0%, containing 5,147 protein-coding sequences, 22 rRNAs, and 86 tRNAs. The ANIs (Average Nucleotide Identity) [calculated by the DFAST pipeline during annotation (6)] between *S. plymuthica* SWSY-3.47 and *S. plymuthica* strains FDAARGOS_895 (GCA_016027675.1) and NBRC-102599 (GCA_001590925.1) were found to be at 97.7% and 97.6%, respectively. It should be noted that the chitinase activity of these two previously sequenced strains is yet to be examined. For the assembled genome of *S. plymuthica* SWSY-3.47, the DFAST annotations did not identify chitinase genes, but we successfully detected six putative chitinase genes by similarity searches. We also found four putative genes encoding lytic polysaccharide monooxygenases, which are intimately involved in chitin degradation (7).

## Data Availability

The genome sequences reported here were deposited in DDBJ under accession number AP035790, and the raw reads were deposited in the Sequence Read Archive (SRA) under BioProject accession number PRJNA1133236 as SRR29747094 run (http://getentry.ddbj.nig.ac.jp/getentry/na/AP035790 https://www.ncbi.nlm.nih.gov/bioproject/1133236 https://trace.ncbi.nlm.nih.gov/Traces/?view=run_browser&acc=SRR29747094&display=metadata).
